# The comorbid network characteristics of anxiety and depressive symptoms among Chinese college freshmen

**DOI:** 10.1186/s12888-024-05733-z

**Published:** 2024-04-19

**Authors:** Jie Luo, Dong-Li Bei, Chuanzhang Zheng, Jie Jin, Chengkui Yao, Jianhua Zhao, Jie Gong

**Affiliations:** 1 School of Psychology, Guizhou Normal University, Guiyang, China; 2 School of Economic and Management, Guizhou Normal University, Guiyang, China; 3 Journal Editorial Department, Guizhou Normal University, Guiyang, China; 4 The School of Psychology and Cognitive Science, East China Normal University, Shanghai, China

**Keywords:** Anxiety, Depression, Comorbidity, Network analysis, Gender differences

## Abstract

**Background:**

This study aimed to investigate the interplay between anxiety and depressive symptoms in Chinese college freshmen using the causal system perspective (CSP), which differs from the traditional common cause perspective (CCP) by providing an alternative explanation by attributing comorbidity to direct interactions among symptoms.

**Methods:**

A convenience sample of 2,082 Chinese college freshmen (39.51% male, *M*_age_ = 18.61) from a normal university completed the Generalized Anxiety Disorder 7-Item Scale (GAD-7) and the Patient Health Questionnaire (PHQ-9). Network analysis was conducted and evaluated as to centrality, stability, node predictability, and bridging features. Moreover, the moderated network model (MNM) was utilized to detect the moderation effects of gender in the comorbidity network.

**Results:**

The network of anxiety and depressive symptoms exhibited stability, characterized by the core symptoms of “restlessness”, “lack of energy”, and “excessive worry about control”, as well as the bridging symptoms of “fearfulness”, “sad mood”, and “irritability”. Notably, the nodes representing “uncontrollable worry” and “difficulty in relaxation” demonstrated the highest predictive power. Gender did not exert any moderating effects on the anxiety and depressive symptom network.

**Conclusion:**

These results reinforce that certain anxiety or depressive symptoms are more central than others, and thus play a more vital role in the comorbid network. These findings highlight underlying potential targeting symptoms to consider in future interventions.

**Supplementary Information:**

The online version contains supplementary material available at 10.1186/s12888-024-05733-z.

## Introduction

Anxiety and depression are the most common mental disorders and numerous studies have reported their high rates of co-occurrence [1, 2]. For instance, a recent meta-analysis pointed out that 42 to 78% of depressed patients suffered from anxiety disorders [2], while another study found that 30 to 63% of individuals with anxiety disorder also met the criteria for comorbid major depressive disorder (MDD) [1]. A large cross-national study involving 27 surveys found consistent patterns of anxiety and depression across different nations (*N* = 74,045), and indicated that approximately 45.70% experienced at least one anxiety disorder among lifetime MDD cases, and that 41.60% had experienced an episode of generalized anxiety disorder within the past year [3]. Therefore, due to the co-occurrence of anxiety and depressive symptoms, it is necessary to treat the two as a complex comorbid system.

Numerous studies have been dedicated to explaining this interplay from various perspectives. Traditionally, most scholars interpret comorbidity under the theory of Common Cause Perspective (CCP), which assumes that mental disorders are a response to latent disorders [4, 5] and are statistically independent symptoms in response to this latent common cause [4]. In this perspective, the co-occurrence or random clustering of different symptoms from a mental disorder are attributed to a latent common cause that cannot be directly observed [5]. Therefore, such methods capture only common variances of all symptoms and overlook information related to the intraindividual development of mental disorders [6, 7].

As an alternative approach, the Cause System Perspective (CSP) considers symptom co-occurrence as a direct interaction between symptoms rather than the latent common cause. This viewpoint suggests that symptoms constitute a mental disorder, rather than simply being its reflection [4, 8]. Network analysis translates conceptual CSP ideas into practical applications by visually depicting the complex associations among symptoms. It seems more reasonable to analyze mental health issues such as anxiety and depression within the CSP framework using network analysis, as patients diagnosed with MDD or comorbid anxiety and depressive symptoms exhibit high heterogeneity [7, 9], making sum-scores susceptible to major errors. Furthermore, due to relatively high predictability within symptoms, the anxiety or depressive symptom network to a large extent is self-determined, but less influenced by external variables [10]. For instance, depressive symptoms can reinforce one another, or the alleviation of one symptom may positively affect other symptoms [6]. Therefore, adopting a symptom-based approach offers an opportunity for further research to explore plausible explanations and insights into the comorbidity between anxiety and depressive symptoms. To note, unless otherwise stated, in this study the terms “symptom”/ “symptoms” specifically pertain to scale item/items rather than clinical diagnose/diagnoses.

In recent years, network analysis has become widely used to investigate the interplay of symptoms associated with mental disorders [11–12], and numerous thorough and comprehensive studies have been conducted on the distinctive features of the network connecting anxiety and depressive symptoms across different groups, including children [13], adolescents [11, 12, 14–18], adults [19–21], and the elderly [22–24]. However, research focused on the symptom network of anxiety and depressive symptoms in university/college freshmen remains limited. Among extant studies, “depressed/sad mood”, “control worry”, and “worry too much” are the three central symptoms most often noted [14–11, 13–16, 19–20, 24], while “sad mood” and “irritable” are the two most recognized bridging symptoms [14–12, 18–19]. However, there is a notable level of inconsistency in the findings derived from these studies. For instance, “fatigue” was considered to be the most central depressive symptom for Chinese female nursing students [12], while “trouble relaxing” was both a core and bridging symptom for UK-, US-, and Australia-based community adults with insomnia [21]. This could be because the data-driven nature of network analysis may yield variable results when investigating diverse populations with different symptoms of anxiety and depression. Additionally, symptom characteristics can also be shaped by social and cultural factors [24]. Therefore, it may not be appropriate to generalize findings based on one specific sample to another subgroup, such as to university/college freshmen.

The college years are a crucial period during which the first onset of various mental health problems (e.g., anxiety and depression) can occur, particularly in first-year college and university students [25]. During this time, students’ lives are undergoing two major changes: transitioning from late adolescence to adulthood [25, 26], and undergoing tremendous change in living environment, such as independent living, adapting to new learning methods, and integrating into new social environments [26–28]. Failure to adjust could lead to a series of mental health problems [29]. Evidence from 19 universities across 8 countries (*N* = 13,984 freshmen) revealed that MDD incidence was the highest during this period, with 21.20% lifetime prevalence and 18.50% in 12-month, followed by GAD at 18.60% lifetime and 16.70% in 12-month [30]. In Hong Kong, China, incidences of depression and anxiety among university freshmen have been found to be 11.90% and 29.10%, respectively [31]. Moreover, a recent systematic review and meta-analysis found that factors such as being female, being a first-year student, and having a family history of mental health are commonly associated with the development of depression among university students [32]. This aligns with the findings of previous studies indicating that females are more likely to experience anxiety and depression during their freshmen year [30, 33]. However, the findings regarding the severity of mental health across gender are inconsistent. For instance, analyses of mediating and moderating effects on Chinese university freshmen (*N* = 1,818) revealed that females exhibited more severe mental health issues as their sense of security decreases [29], whereas in a cohort study (2005–2011; *N* = 13,085), male participants reported poorer levels of mental health than females [34]. Meanwhile, longitudinal studies have revealed an inverse relationship between students’ year of study and psychological health problems [31, 35], and mental health issues among freshmen can lead to long-term adverse outcomes in later adulthood [26]. Therefore, it is imperative to examine the features of anxiety and depressive symptoms in college/university freshmen from a network perspective to provide more pertinent information for the development of intervention strategies.

This study is first to explore the comorbid relationships between anxiety and depressive symptoms using a network model in a non-clinical sample of Chinese college freshmen. In consideration of previous research, this study aimed to: (1) examine the structure and characteristics of the network of anxiety and depressive symptoms in college freshmen, (2) evaluate the stability of the symptom network, and (3) explore the possible gender differences in the network. We expected that “sad mood,” “control worry,” and “worry too much” would be the most central symptoms, and that “sad mood” and “irritable” would be the bridging symptoms in the network [19]. In addition, we expected that the edges and centrality indices would be stable, as seen in previous studies. Finally, considering past findings of no differences in the anxiety and depression networks between genders [11, 22] as explored by network comparison test (NCT), we expected the same outcome in this study.

## Methods

### Participants

Given that symptoms exhibit important similarities across different levels of disease [36], the current study sample included freshmen with anxiety and depressive symptoms ranging from normal to abnormal levels to gain a comprehensive understanding of comorbid anxiety and depressive symptoms. Inclusion criteria for participants were as follows: (1) full-time freshmen only, and (2) proficiency in Chinese and ability to understand the assessment materials. Exclusion criteria for participants were: (1) failure to answer at least 20% of the main questionnaire, (2) data with regular responses (e.g., choosing only one answer, Z-shaped, or S-shaped answers), and (3) data with unreasonable responses (e.g., most values were outside the given range). The study sample initially comprised 2,088 participants, all studying at a normal university in Guizhou Province, China, in September 2023. After excluding six data points with missing values exceeding 20%, 11 outliers were excluded on the first item of the GAD-7 and the last item of PHQ-9. All missing values were imputed using full information maximum likelihood (FIML), and the final dataset comprised 2,082 participants, with the average age of participants being 18.61 (*SD* = 0.86). Among the final participant data, 1,251 (60.06%) were female, 822 (39.51%) were male, and nine (0.04%) did not report their gender.

### Procedure

All participants provided their written informed consent prior to participation in this study, and were fully briefed on the survey’s purpose, confidentiality, anonymity, and voluntary nature. Data collection took place in a formal classroom setting on a regular school day. Participants took approximately 10 min to complete the questionnaire. All research assistants involved in the data collection underwent professional training. This study was approved by the Institutional Review Board (or Ethics Committee) of School of Psychology of Guizhou Normal University (GZNUPSY.N.202208E [0027]).

### Measures

#### Generalized anxiety Disorder-7 (GAD-7)

The GAD-7 [37] is a well-established psychometric instrument and has been widely utilized to assess respondents’ severity of anxiety over the previous two weeks. It is a unidimensional scale and consists of seven items, each one rated using a four-point Likert scale: 0 = “not at all”, 1 = “several days”, 2 = “more than half the days”, and 3 = “nearly every day”. A cutoff score of 10 has been established for this scale, demonstrating a sensitivity of 86.20% and specificity of 95.50% [37]. The Chinese version of the GAD-7 has been used successfully in various Chinese settings [38, 39]. The Cronbach’s α for the GAD-7 in this study was 0.90.

#### Patient Health Questionnaire-9 (PHQ-9)

The PHQ-9 [40] is a reliable and effective self-report questionnaire used to measure the severity of one’s depressive symptoms in both general research and clinical settings. The PHQ-9 consists of nine items, with each item rated on a four-point Likert scale ranging from 0 = “not at all” to 3 = “nearly every day”. Previous studies have demonstrated the validity and utility of the Chinese version of the PHQ-9 in screening for depression among both Chinese adolescents [41] and the general population [42]. A cutoff of seven exhibits an equal sensitivity and specificity rate of 86% [42]. The Cronbach’s α for the current study was 0.86.

### Statistical analysis

Descriptive statistics, such as socio-demographic distribution, mean scores, standard deviations, normality, and inferential statistics including a series of *t*-tests and chi-square tests, were conducted using JASP version 0.16.4.0 [43].

The undirected network was estimated via Gaussian Graphical Model (GGM) [44] using R package *qgraph* [45]. The graphic least absolute shrinkage and selection operator (GLASSO) [46] algorithm was used to regularize the results to obtain a sparse network. The tuning parameter was set to 0.50 to balance the sensitivity and specificity of the true network edges [47].

The symptom network was characterized using Expected Influence (EI), bridging Expected Influence (bEI), and node predictability with R package *qgraph* [45], *networktools* [48], and *mgm* [49], respectively. A higher EI value indicates a greater importance of the node within the network. A higher bEI value suggests an increased risk of node contagion to other communities, with one-step and two-step bEI referring to direct and indirect bridge effects between a node and nodes outside its community, respectively. The cutoff for identifying bridging symptoms was set at the 80th percentile [50]. Predictability was measured by the proportion of the explained variance, ranging from 0 to 1, with 0 indicating the absolute inability to predict one node by others in the network, and 1 indicating perfect prediction abilities [10].

The accuracy and stability of the network were examined with R package *bootnet* [51]. A non-parametric bootstrapping method was utilized to estimate 95% confidence interval (CI) of each edge weight. Then, EI and bEI stabilities were quantified using the correlation stability coefficient (CS) via the case-dropping subset bootstrap approach, by which the CS value should not fall below 0.25 and preferably be above 0.50 [51]. Finally, bootstrapped difference tests were calculated to determine the significant difference between each pair of edge weights.

To examine the gender differences in the network of anxiety and depressive symptoms, a moderated network model (MNM) was employed using R Package *mgm* [52]. MNM enables the comparison of network differences among multiple groups within a unified model. The optimal tuning parameter λ was determined by minimizing the EBIC and the hyperparameter γ = 0.50 by setting lambdaSel = “EBIC” and lambdaGam = 0.50 [52].

## Results

### Descriptive statistics

The sample used in this study included 2,082 Chinese college freshmen with varying levels of anxiety and depressive symptoms. Among them, 217 (10.42%) and 690 (33.14%) reported experiencing anxiety and depression, respectively. Females exhibited higher vulnerability to anxiety and depression as compared to males (GAD-7: 134 females vs. 83 males; PHQ-9: 424 females vs. 261 males). There were no significant differences across gender in terms of anxiety and depression severity (GAD-7 ≥ 10: χ^2^ = 0.56, *p* = 0.57; PHQ-9 ≥ 7: χ^2^ = 1.38, *p* = 0.17). See Tables 1 and 2 for more details.

**Table 1:** Socio-demographic and mental health features of Chinese college freshmen

	Males	Females	χ^2^/*t*	*p*-value
	(*N* = 822)	(*N* = 1,251)		
Residence				
- City	221 (10.61%)	319 (11.38%)	0.56	0.46
- Rural	588 (20.99%)	916 (44.00%)		
Only-child status				
- Yes	151 (7.30%)	151 (7.30%)	16.29	< 0.001
- No	656 (31.51%)	1084 (52.07%)		
Ethnicity				
- Han	470 (22.57%)	750 (36.02%)	1.66	0.20
- Other minority	351 (16.86%)	498 (23.92%)		
GAD-7				
- GAD-7 ≥ 10	83 (3.99%)	134 (6.44%)	0.20	0.66
- GAD-7 < 10	739 (35.49%)	1,117 (53.65%)		
PHQ-9				
- PHQ-9 ≥ 7	261 (12.54%)	424 (20.37%)	1.03	0.31
- PHQ-9 < 7	561 (26.95%)	827 (39.72%)		
GAD-7 ≥ 10, mean (*SD*)	13.29 (3.21)	13.05 (2.89)	0.56	0.57
PHQ-9 ≥ 7, mean (*SD*)	10.75 (4.24)	10.32 (3.61)	1.38	0.17

*Notes*: GAD-7 = Seven-Item Generalized Anxiety Disorder scale, PHQ-9 = Nine-Item Patient Health Questionnaire, χ^2^ = chi-square tests for residency, only-child status, ethnicity, GAD-7, and PHQ-9; *t* = independent *t*-test result for mean values of GAD-7 ≥ 10 and PHQ-9 ≥ 7

**Table 2:** Mean scores, standard deviations, normality, item abbreviation, and predictability of each symptom noted in the GAD-7 and PHQ-9

	Item abbreviation	*M*	*SD*	*S-W*	Predictability
Anxiety (GAD-7)					
GAD1: Feeling nervous, anxious, or on edge	Nervous	0.87	0.68	0.77^***^	0.51
GAD2: Not being able to stop…	Control worry	0.68	0.74	0.77^***^	0.61
GAD3: Worrying too much about…	Worry too much	0.82	0.77	0.80^***^	0.58
GAD4: Trouble relaxing	Relax	0.60	0.75	0.74^***^	0.60
GAD5: Being so restless that it’s hard to sit still	Restless	0.46	0.67	0.68^***^	0.58
GAD6: Becoming easily annoyed or irritable	Irritable	0.62	0.73	0.75^***^	0.55
GAD7: Feeling afraid as if something awful…	Afraid	0.46	0.70	0.67^***^	0.49
Depression (PHQ-9)					
PHQ1: Little interest or pleasure…	Anhedonia	0.78	0.78	0.80^***^	0.43
PHQ2: Feeling down, depressed, or hopeless	Sad mood	0.56	0.68	0.73^***^	0.49
PHQ3: Trouble falling or staying asleep…	Sleep	0.63	0.81	0.74^***^	0.32
PHQ4: Feeling tired or having little energy	Energy	0.84	0.82	0.81^***^	0.50
PHQ5: Poor appetite or overeating	Appetite	0.69	0.80	0.77^***^	0.36
PHQ6: Feeling bad about yourself…	Guilt	0.54	0.75	0.71^***^	0.45
PHQ7: Trouble concentrating on things…	Concentration	0.70	0.82	0.77^***^	0.37
PHQ8: Moving or speaking so slowly…	Motor	0.37	0.66	0.60^***^	0.45
PHQ9: Thoughts that you would be…	Suicide	0.15	0.48	0.36^***^	0.32

*Notes: SD* = standard deviation, *S-W* = Shapiro-Wilk test, GAD-7 = Seven-Item Generalized Anxiety Disorder scale, PHQ-9 = Nine-Item Patient Health Questionnaire, ***p < 0.001

### Network Analysis

**Fig. 1:**
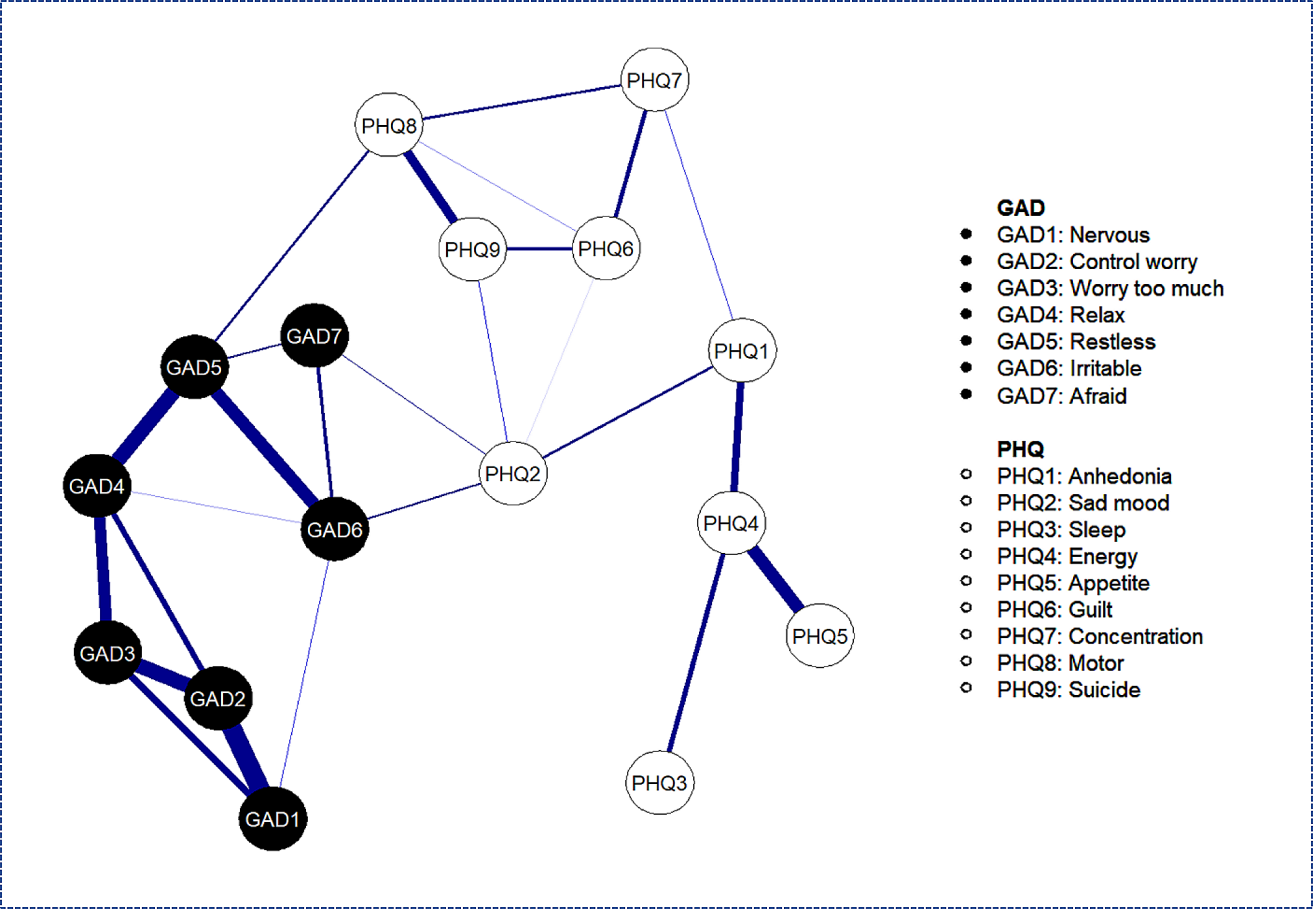
Network of anxiety and depressive symptoms among Chinese college freshmen as revealed by the GLASSO Algorithm. *Notes*. Blue edges indicate positive partial correlations between nodes, while the thickness of the edge reflects the strength of the correlation. Edges representing small partial correlations, such as the red ones indicating negative correlations, were eliminated from the network as GLASSO shrunk them to zero

**Fig. 2:**
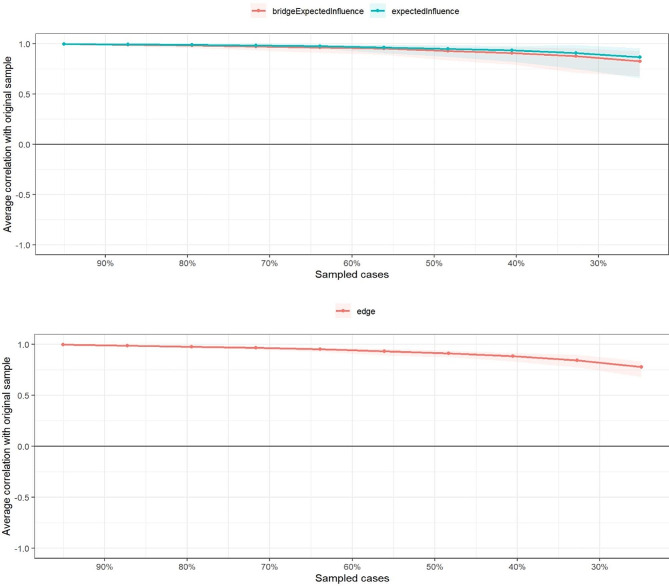
Correlation stability coefficients (CS) for the nodes’ centrality and edge weights

Figure 1 presents the network structure of anxiety and depressive symptoms. The mean weight of the network was 0.05, with 25% of all 120 network edges being zero. Among anxiety symptoms, the five strongest edges were between “nervous” and “control worry” (edge weight = 0.25), “control worry” and “worry too much” (edge weight = 0.21), “relax” and “restless” (edge weight = 0.21), “restless” and “irritable” (edge weight = 0.19), and “worry too much” and “relax” (edge weight = 0.18). Additionally, among depression symptoms, the strongest edge was observed between “energy” and “appetite” (edge weight = 0.19). Bootstrapped 95% CI results suggested that the assessment of the edge weights were relatively reliable (see Appendix A1), and all of the above-noted strongest edges were significantly larger than any other edges, according on the edges difference test (see Appendix A2). The CS of the edge was 0.75 (see Fig. 2).

**Fig. 3:**
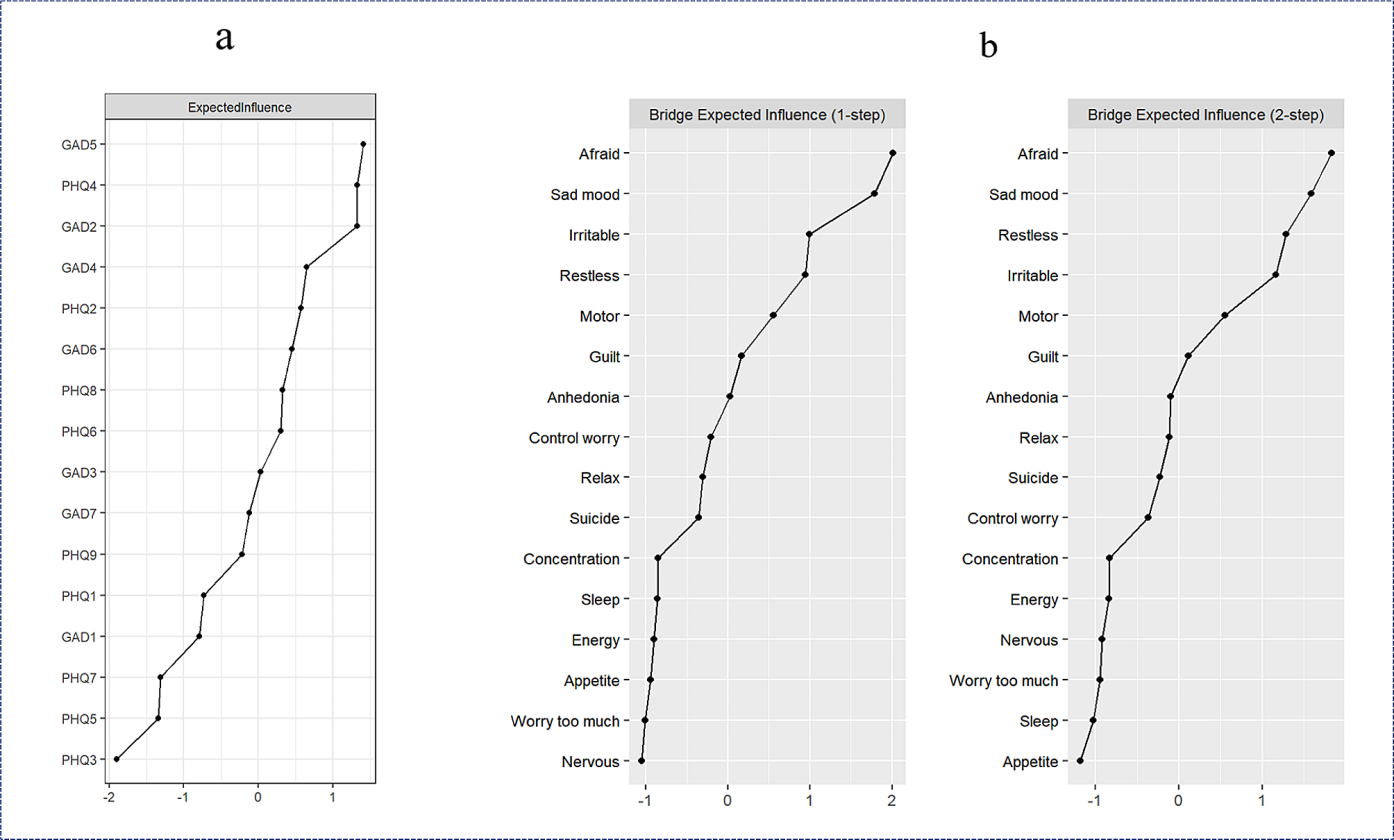
The centrality and bridging characteristics for all nodes of the symptom network

As Fig. 3a shows, EI indicators of all symptoms from the estimated network identified the three most central symptoms– “restless”, “energy”, and “control worry”– indicating that these three symptoms were the most associated symptoms. Symptoms of depression related to “concentration”, “appetite”, and “sleep” all exhibited the lowest EI values, suggesting that these symptoms were the least interconnected symptoms. Results of the bootstrapped difference test for EI revealed a significant difference between these central symptoms, and 70 to 87% between those and the other symptoms (see Appendix A3). Additionally, the expected influence CS was 0.75, indicating the adequate stability of EI (see Fig. 2).

Figure 3b presents the 80th percentile of the one-step and two-step bEI symptoms. The graph depicting anxiety symptom “afraid” and depression symptom “sad mood” indicates that these two symptoms had the strongest ability to increase risk from one community to the other, both directly and indirectly. Anxiety symptom “irritable” (one-step) and “restless” (two-step) demonstrated the ability to increase risk of contagion to depression directly and indirectly, respectively. The CS was 0.67, suggesting that the node bEI evaluation was quite stable (see Fig. 2). The bootstrapped difference test revealed a significant difference between these bridging symptoms and 47 to 80% of the other symptoms (see Appendix A4). Finally, meaningful bridge routes were also found between the nodes of “restless” and “motor” (weight = 0.11), “irritable” and “sad mood” (weight = 0.10), and “afraid” and “sad mood” (weight = 0.10; see Fig. 1).

Table 2 also revealed that the average predictability (AP) of nodes within the entire network was 0.48, with anxiety nodes exhibiting a higher AP value of 0.56 compared to the depression nodes, at 0.41. Notably, the anxiety symptoms of “control worry” (0.61), “relax” (0.60), “restless” (0.58), and “worry too much” (0.58) displayed the highest levels of predictability among all nodes in the network. See Table 2 for more detailed information.

**Fig. 4:**
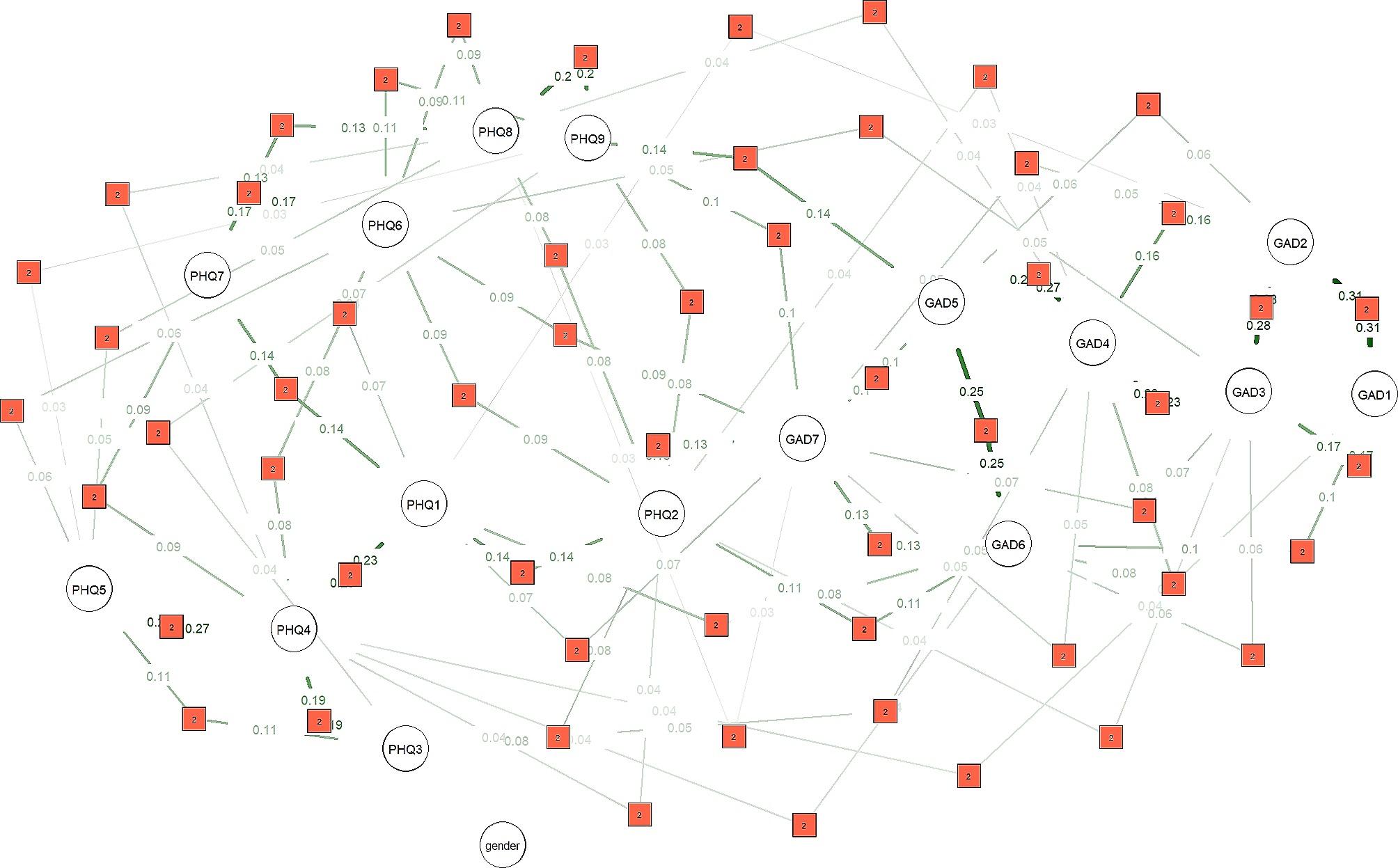
Network structure of anxiety and depressive symptoms as moderated by gender, with the red box between two nodes representing pairwise interactions

Finally, Fig. 4 showed the network structure of anxiety and depressive symptoms in relation to gender. The results indicated that the network was remarkably stable and not regulated by gender, as there were no significant connections between gender and any of the symptoms of anxiety or depression. Moreover, the network structure appeared relatively simple, consisting solely of pairwise interactions. The strongest pairwise interactions for anxiety symptoms were found between “nervous” and “control worry” (0.31), “control worry” and “worry too much” (0.28), “relax” and “restless” (0.27), “restless” and “irritable” (0.25), and “worry too much” and “relax” (0.23), and, for depression symptoms, “energy” and “appetite” (0.27), “anhedonia” and “energy” (0.23), and “motor” and “suicide” (0.20). These pairwise interactions could be explained through linear regression, such as the association between “nervous” and “control worry” (0.31). When the node “nervous” was increased by one unit, the symptom “control worry” increased by 0.31 units while all other conditions remained constant.

## Discussion

This study is the first to examine the comorbidity of anxiety and depressive symptoms among Chinese college freshmen at the symptom level using a network approach. The results of this study indicate that the symptoms have different positions and identities within the network, suggesting that identifying node attributes can benefit disease interventions. For example, symptoms such as “restless”, “energy”, and “control worry” were all found to be positioned more central than the others, suggesting that intervening in these symptoms specifically could yield positive effects. Meanwhile, interventions focused on nodes with high predictability (e.g., “control worry”, “relax”, “restless”, and “worry too much”) can be accomplished by intervening in their neighboring nodes. Some symptoms exhibited more cross-symptom attributes (e.g., “restless” and “motor”, “irritable” and “sad mood”, and “afraid” and “sad mood”), which could represent advancements in explaining and intervening in comorbidities. Finally, this study also revealed that gender did not influence the overall network structure.

### Network of anxiety and depressive symptoms

This study found that all the strongest edge links were yielded within symptoms constituting a certain disorder, which is consistent with previous studies [4, 12, 19, 24]. Previous studies have used a variety of samples to identify the majority of the network edges, such as Chinese female nursing students [12], psychiatric samples [4], and migrant Filipino domestic workers [19]. In this study, the connections between “nervous” and “control worry”, “control worry” and “worry too much”, “worry too much” and “relax”, as well as “energy” and “appetite” were relatively stable across different contexts. These edges may reflect the presence of a specific pattern or pathological mechanism among symptoms, and provide further hints as to the pathogenesis or etiology of the disease. This study also identified another strong connection between the anxiety symptoms of “relax” and “restless.” This connection was caused largely by university/college freshmen facing new environments and challenges, such as adapting to new lifestyles and social interactions [29].

Each node’s EI may play an important role in identifying the symptoms that activate or maintain psychopathological networks, and as well as in finding potential targets for intervention [12, 14]. The present study found that the nodes for “restless”, “control worry”, and “energy” were the most central. “Control worry” refers to one’s inability to stop or control their worrying, which has been identified consistently as a central symptom in existing studies on anxiety and depressive symptom networks [14, 20–24]. Thus, the identification of this symptom was as expected. It was, however, unexpected that both “restless” and “energy” also demonstrated high centrality as anxiety symptoms. This could be due to changes in the students’ environment, academic pressures, or intricate interpersonal interactions, all of which might lead to that adaptive sleep problems, which has emerged as a common issue among college freshmen [53], consequently elevating their risk of less energy and restlessness. Additionally, somatization of distress is common among Asian populations [12, 19], which may contribute to “fatigue” and “restless” being prominent central symptoms. This may reflect the unique characteristics of Chinese college freshmen. In summary, the three central symptoms of “restless”, “irritable”, and “control worry” were shown to be critically instrumental in activating and maintaining the network of anxiety and depressive symptoms among Chinese college freshmen.

A node’s bEI may help identify bridging symptoms that play pivotal roles in the development and maintenance of the co-occurrence of mental disorders. This study identified the three symptoms with the highest bEI values: “afraid” and “irritable” from anxiety symptoms, and “sad mood” from depression symptoms. The latter two were identified as bridging symptoms as expected, which is consistent with the findings of previous studies [12, 14, 19, 23]. However, we also identified “afraid” as another bridging symptom, which could be attributed to the characteristics of our specific sample. Freshmen may be experiencing worries about their new environments, interpersonal relationships, or academic performance. Additionally, these three bridge symptoms form two bridge routes: “irritable” and “sad mood”, and “afraid” and “sad mood”. Research has shown that there is a bidirectional predictive relationship between anxiety and depression [54, 55] which, in combination with the findings of the current study, implies that despite the relatively weak connections, these two pathways play a crucial role in the development of comorbidity between anxiety and depressive symptoms. In other words, nodes with a higher bEI may be more effective in preventing or reducing comorbid symptoms [50].

The average node predictability was 0.48, suggesting that the present network was moderately self-determined [10]. The predictability of nodes can reflect the controllability of a network and determine the effectiveness of planned treatment [10]. In this study, “control worry”, “relax”, “restless”, and “worry too much” all exhibited high predictability values. This could be a result of these symptoms measuring distinct yet interrelated concepts or capturing analogous constructures. Symptoms with high predictability can be controlled through intervention on their neighboring nodes. Meanwhile, for nodes with low predictability, such as “suicide”, “appetite”, and “sleep”, effective interventions may be attributed to the low variance of these nodes with others in the network, and can be either controlled directly or focused on factors outside the network, such as additional symptoms or biological and environmental variables [10, 12].

Finally, this study found that gender had no effect on the entire network structure, which is also consistent with the findings of previous studies [11, 22]. This suggests that interventions for anxiety and depressive symptoms can target key nodes in the network without giving excessive consideration to gender factors. Furthermore, while studies have indicated that women have a greater vulnerability towards anxiety and depression, the present study found no statistically significant gender-based disparities in terms of either quantity or severity, contradicting previous findings. Given that freshmen face a changing environment, the lack of gender-based effects could be partly attributed to gender differences in biological susceptibility, self-esteem, and exposure to stress [56], or it could be due to the cultural background of Chinese collectivism. Moreover, our moderated network model revealed that the entire network was comprised of only pairwise interactions among symptoms, without any three-way or higher interactions. This suggests that the anxiety and depressive symptom network among Chinese freshmen was not excessively complex, and that interventions targeting symptoms with strong pairwise interactions can be expected to effectively alleviate anxiety and depressive symptoms in Chinese college freshmen.

### Implications

This study is the first to utilize network analysis to examine the relationships between anxiety and depression symptoms in Chinese college freshmen. The findings offer valuable insights for the development of clinical interventions targeting symptoms of anxiety and depression in freshmen. The central symptoms identified in this study, specifically “restless”, “energy”, and “control worry”, are able to activate and sustain the psychopathic network. Meanwhile, bridging symptoms like “afraid”, “irritable”, and “sad mood” can activate and maintain other disorders via bridging pathways (e.g., between “afraid” and “sad mood”, as well as between “irritable” and “sad mood”), making them clinically transdiagnostic. Intervention strategies should focus on the most highly predictable symptoms found in the network, such as “control worry”, “relax”, “restless”, and “worry too much”. Meanwhile, effective intervention for symptoms with low predictability (e.g., “sleep” and “suicide”) may target factors outside the network or directly address these specific symptoms. Finally, based on these findings from the moderated network analysis, it is clear that the relationship between anxiety and depressive symptoms in freshmen is not excessively complex. This suggests that timely interventions should lead to positive outcomes.

### Limitations and future directions

Despite its contributions to the literature, this study nonetheless has some limitations. First, the results of this study were derived solely from self-report data rather than from structural clinical interviews. Future studies should validate these findings by utilizing structural rating tools in clinical freshmen samples. Second, making causal inferences from an undirected network is challenging. Future studies should employ experimental or prospective designs to examine the causal or longitudinal relationship between anxiety and depressive symptoms. Third, the outcomes of the network may vary depending on the tools used [57]. Non-DSM symptoms have been shown to be more central compared to DSM symptoms [58]. Future studies should broaden the range of testing and explore more central symptoms rather than focusing solely on specific DSM symptoms (e.g., by using the GAD-7 or the PHQ-9).

## Conclusions

The primary aim of this study was to identify the network characteristics of anxiety and depressive symptoms among Chinese college freshmen. The most central symptoms were the anxiety symptoms of “restless” and “control worry”, and the depression symptom of “energy”. The bridging symptoms identified were “afraid”, “irritable”, and “sad mood”. Furthermore, although the connections of the bridging pathways were relatively weak, their presence still suggests that these connections might contribute to the development of comorbidity. Our findings also showed that node predictability is also crucial for the development of intervention strategies which target the overall network. Finally, the symptom network of anxiety and depressive symptoms in Chinese college freshmen was not excessively intricate, thus timely intervention should lead to positive outcomes.

### Electronic supplementary material

Below is the link to the electronic supplementary material.

Supplementary Material 1

## Data Availability

The data supporting the conclusions of this study are available upon request to the corresponding author, Jie Luo.
