# Supplementary material for: The comorbid network characteristics of anxiety and depressive symptoms among Chinese college freshmen
Source: BMC Psychiatry. 2024 Apr 19;24:297. doi: 10.1186/s12888-024-05733-z (PMC11027377; doi:10.1186/s12888-024-05733-z)
Supplement: Supplementary file 1 — Supplementary Material 1 [file 12888_2024_5733_MOESM1_ESM.docx]

**Supplementary Material**

**Appendix**

The following figures provide results for network edge weights and edge differences using non-parametric bootstrapping method.


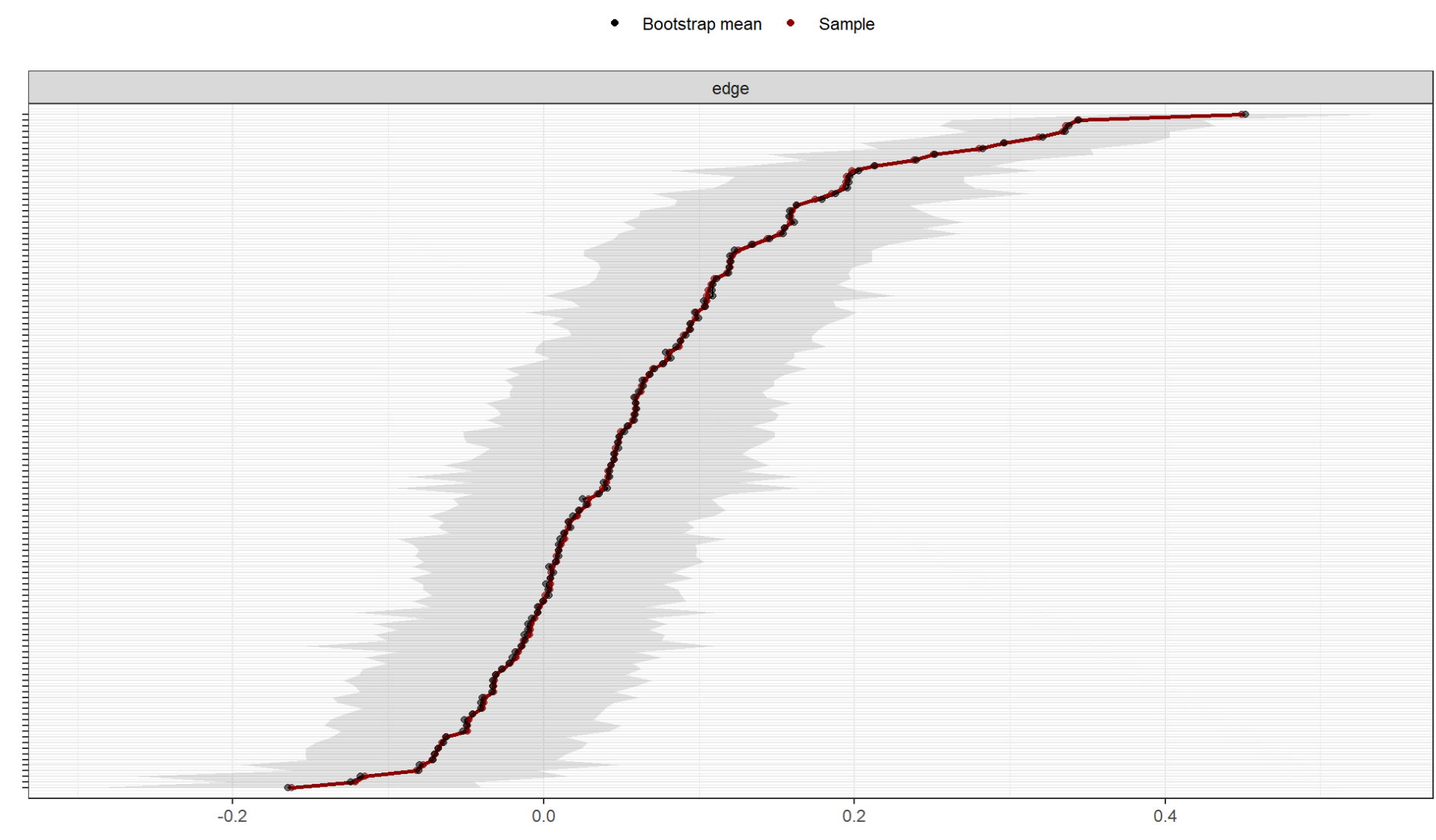


**A1** 95% bootstrapped confidence interval with non-parametric bootstrapping method


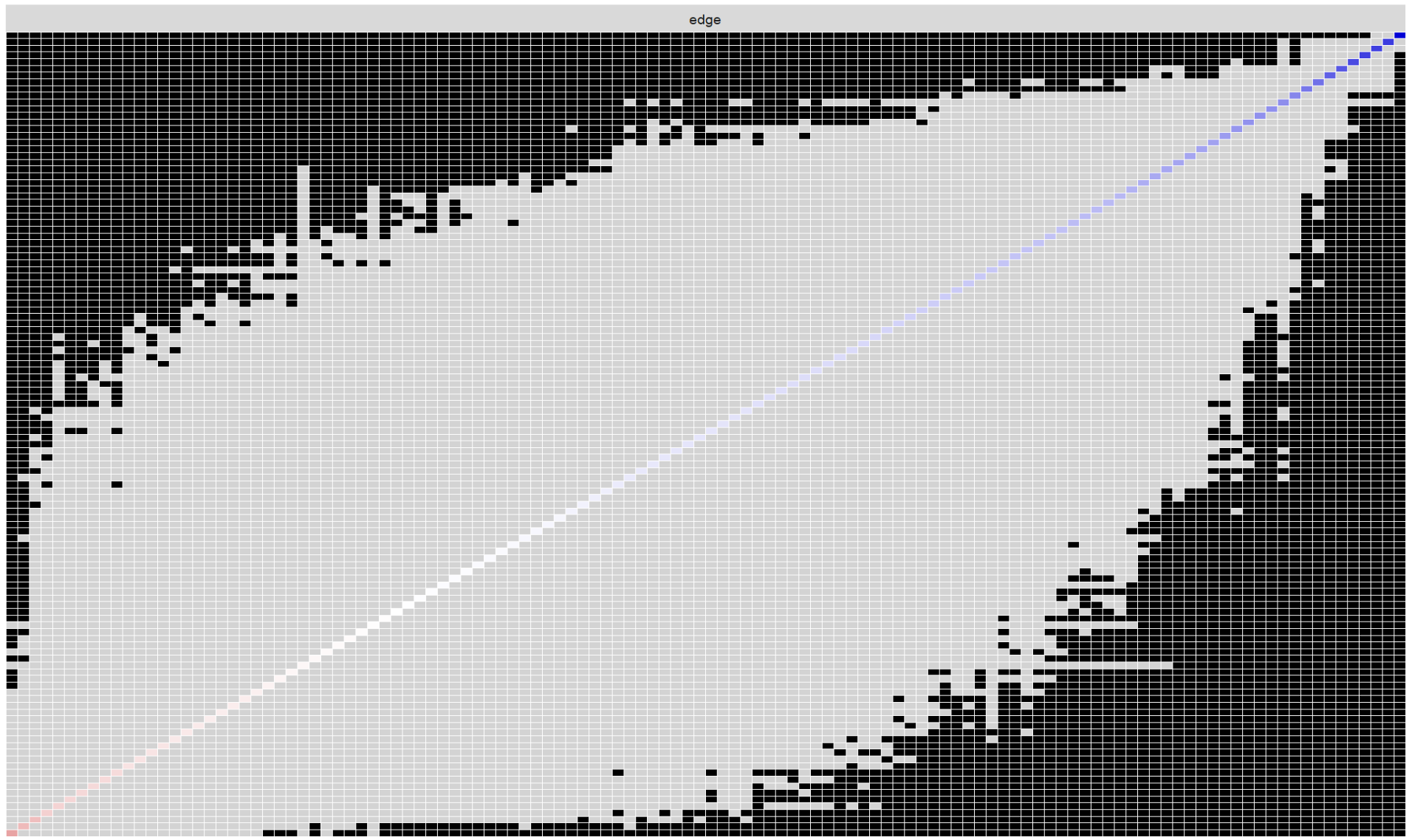


**A2** Bootstrapped difference tests for edges among symptoms


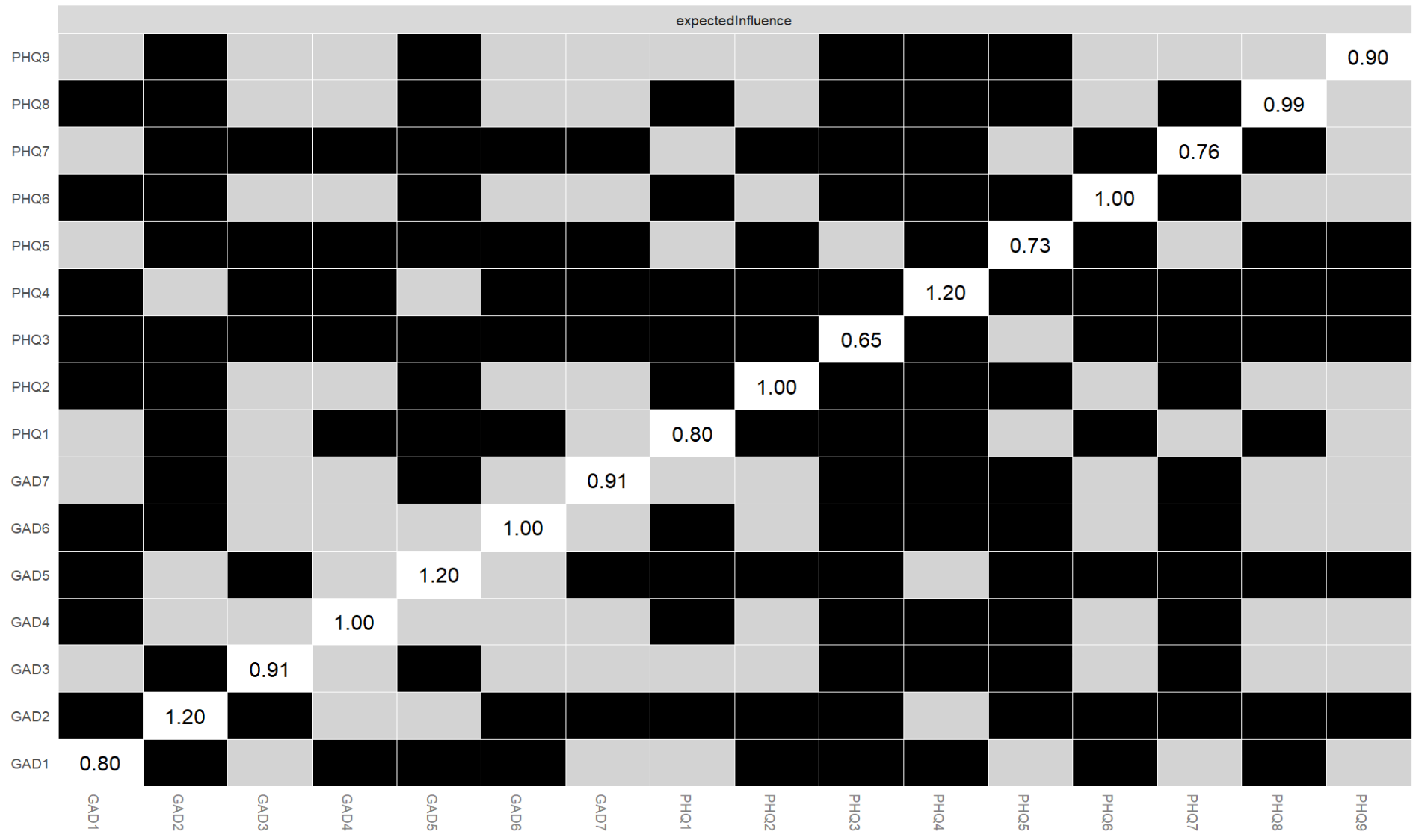


**A3** Bootstrapped difference tests for Nodes’ expected influence


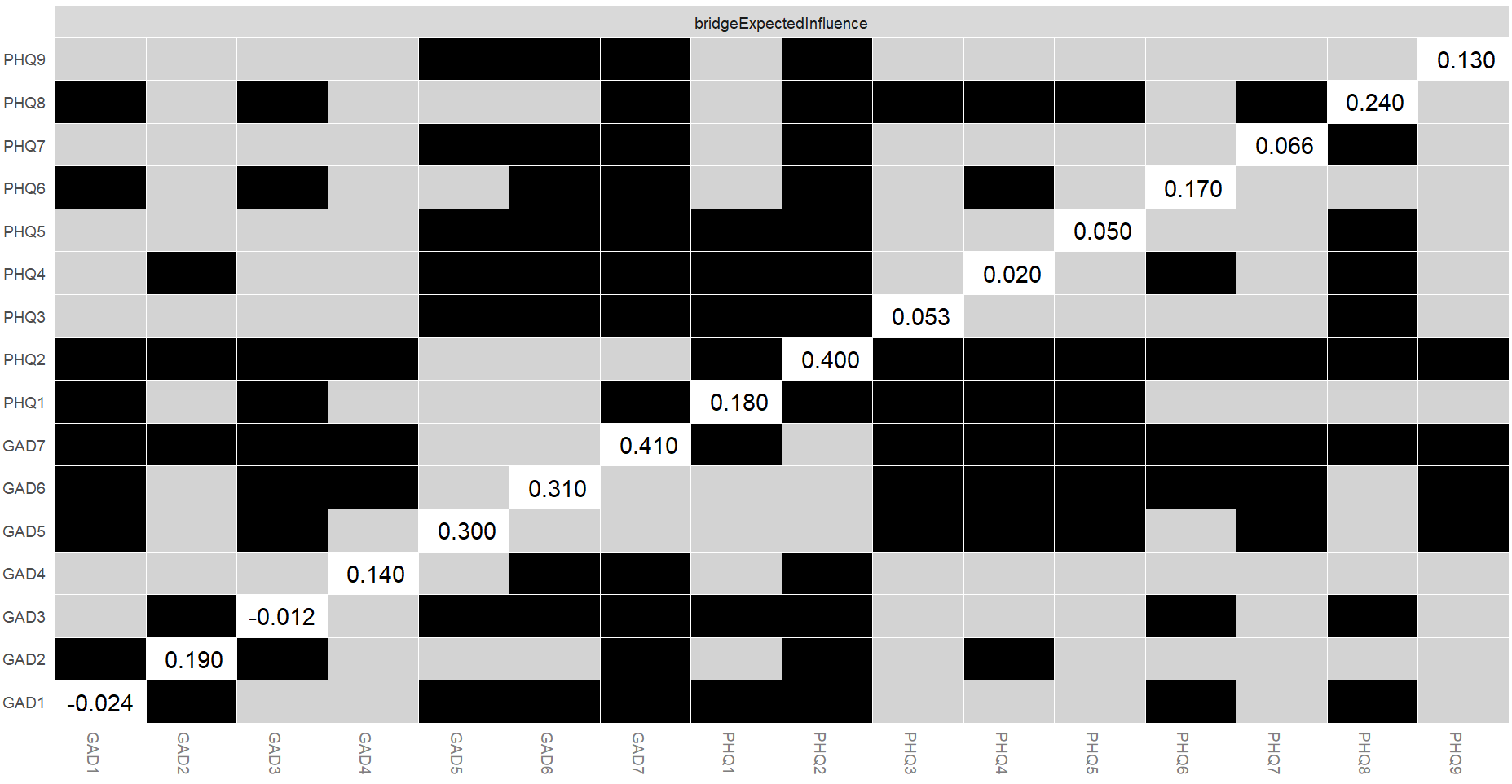


**A4** Bootstrapped difference tests for bridging expected influence
